# Influence of Gender and Age of Brown Seaweed (*Fucus vesiculosus*) on Biochemical Activities of Its Aqueous Extracts

**DOI:** 10.3390/foods11010039

**Published:** 2021-12-24

**Authors:** Diogo Nunes, Rebeca André, Asma Ressaissi, Bernardo Duarte, Ricardo Melo, Maria Luísa Serralheiro

**Affiliations:** 1 BioISI—Biosystems & Integrative Sciences Institute, Faculdade de Ciências, Universidade de Lisboa, 1749-016 Lisboa, Portugal; diogomnuness@gmail.com (D.N.); rebeca.esperanca@gmail.com (R.A.); aressaissi@fc.ul.pt (A.R.); 2 Marine and Environmental Sciences Centre (MARE), Faculdade de Ciências, Universidade de Lisboa, 1749-016 Lisboa, Portugal; baduarte@fc.ul.pt (B.D.); ramelo@fc.ul.pt (R.M.); 3 Departamento de Biologia Vegetal, Faculdade de Ciências, Universidade de Lisboa, Campo Grande, 1749-016 Lisboa, Portugal; 4 Department of Chemistry and Biochemistry, Faculdade de Ciências, Universidade de Lisboa, Campo Grande, C8 bldg, 1749-016 Lisboa, Portugal

**Keywords:** *Fucus vesiculosus*, phlorotannin, antioxidant activity, acetylcholinesterase, heavy metals, LC-HRMS/MS

## Abstract

*Fucus vesiculosus* L. is a common coastal brown seaweed associated with various benefits to human health due to its phenolic content and nutrients and is used as food through different methods of consumption. This study aims to evaluate the influence of the seaweed’s gender and growth stage on different types of biological activities as well as its chemical constitution and elements present. Akin to food preparation, aqueous extracts of the seaweed were prepared at 25 °C (salad) and 100 °C (soup). Biological activities were determined by measuring total phenol content (TPC), antioxidant activity and inhibition of acetylcholinesterase (AChE). Liquid Chromatography High Resolution Mass Spectrometry (LC-HRMS/MS) was used for compound identification, and elemental analysis was carried out by using Total Reflection X-ray Fluorescence Spectrometry (TXRF). Older females and males had higher TPC compared to the new ones at 100 °C. Antioxidant activity depended on the extraction temperature but was higher for the youngest male at 100 °C. AChE inhibitory activity was higher for older males at 25 °C, but at 100 °C it was higher for older females. Primary metabolites and various phloroglucinol were the main compounds identified. Additionally, since this seaweed is often harvested in estuarine systems with high anthropogenic impacts, its safety was evaluated through the evaluation of the sample’s metal content. The heavy metals detected are within the limits established by various regulating entities, pointing to a safe food source.

## 1. Introduction

In the last few years, natural products, particularly plant-based ones, have been experiencing an increasing interest both due to their inherited properties and as an alternative to synthetic supplements [1]. *Fucus vesiculosus* L. is a perennial species classified within the Ocrophyta division, class Phaeophyceae—brown seaweeds—generally distributed alongside the North Atlantic and temperate coastal areas [2], and it is sought after as a result of their low-fat content [3], rich (>40%) polyunsaturated fatty acids [4], high level of minerals [5] and high contents in polyphenols and non-digestible polysaccharides [6].

Exclusive to brown seaweeds, phlorotannins, an understudied specific type of polyphenols, are polymeric derivatives of phloroglucinol [7] that have been linked with higher antioxidant activities [8]. In the normal process of aerobic cellular metabolisms, radicals such as Reactive Oxygen Species (ROS) or Reactive Nitrogen Species (RNS) are formed as by-products; however, a debilitated antioxidant system within the organism may cause an overproduction of these reactive species [9]. As a consequence, oxidative stress can result in several chronic and degenerative diseases, such as cancer [10]; cardiovascular diseases (CVD), such as atherosclerosis, ischemia, hypertension or cardiomyopathy [11]; neurological diseases (Alzheimer’s Disease, Parkinson’s disease, multiple sclerosis and amyotrophic lateral sclerosis) [12,13]; pulmonary diseases (asthma and chronic obstructive pulmonary disease) [14,15]; rheumatoid arthritis [16]; renal diseases (e.g., glomerulonephritis or tubulointerstitial nephritis [17]; ocular diseases (e.g., cataracts) [18]; and pre-eclampsia in prenatal medicine [17].

Another potential aspect of *F. vesiculosus* resides in its capability to alleviate Alzheimer’s Disease (AD) symptoms. AD is a type of brain disease that damages and destroys neurons. In the early stages, its symptoms are easily confused with normal signs of ageing; however, as the disease progresses, it starts to affect cognitive functions, such as thinking and planning, and it evolves into affecting the most basic bodily functions to the point where the patient is completely dependent on a caretaker [19]. To this day, AD has no known treatment able to stop the damage to neurons associated with it; therefore, current treatments aim at alleviating AD’s symptoms. Extracts rich in phlorotannins have been shown to exhibit inhibitory effects on the activity of acetylcholinesterase (AChE) [20,21], an enzyme that catalyzes the breakdown of acetylcholine (ACh) [22], which in an individual with AD is usually found in much lower quantities [23]. This molecule (ACh) mediates neurotransmission in the brain and it is thought that a loss of cholinergic functions associated with it in the Central Nervous System contributes greatly to the loss of cognitive functions linked to AD [23].

In another light, the high mineral content of *F. vesiculosus* is promising due to its inherent nutritional value associated with *F. vesiculosus* composition, in particular, Na, K, I, Ca, Fe, Mg, Cu, Zn and Mn [3]. In addition to studying this seaweed’s beneficial elements, analyzing its mineral constitution is also important for detecting the presence of harmful heavy metals (e.g., Hg, As and Cd) for human consumption, as brown seaweeds are known for their ability to bioaccumulate them. Moreover, *Fucus vesiculosus* can also function as a bioremediator [24] in habitats that are polluted with harmful toxic elements, such as heavy metals, or as a bioindicator to verify the adaptability of the species in different habitats [4].

Thus, considering the abovementioned qualities and traits of *F. vesiculosus,* the present study aimed to elaborate on a biochemical and mineral characterization of *F. vesiculosus* harvested from the Tagus Estuary. In particular, as *F. vesiculosus* constitution may vary depending on its habitat, environmental conditions and maturity [3], the influence of the growth stage and gender of this seaweeds was studied in different aspects: (i) Total Phenolic Content (TPC); (ii) antioxidant activity and its relation with the TPC; (iii) composition of *F. vesiculosus* throughout its development and between genders; (iv) inhibition towards AChE to study the potential of this seaweed in the treatment of symptoms related to AD; and (v) mineral composition both in *F. vesiculosus* beneficial components and the presence of heavy metals in its constitution. Ultimately, through the characterization of *Fucus vesiculosus* as described, this study seeks to find the optimal conditions for its harvesting depending on the stage of growth and gender that are deemed best for the properties studied.

## 2. Materials and Methods

### 2.1. Seaweeds Material

Sampling of *F. vesiculosus* L. was performed in the Tagus Estuary (38°46′56.2″ N 9°05′27.7″ W) on 10 October 2018. The seaweed samples were collected and classified immediately—four thallus length classes of *F. vesiculosus* were previously established (<20 cm; 20–25 cm; 25–30 cm; >30 cm size)—corresponding to proxies of thallus age with the 1st class being same year recruits and successive longer thalli being older cohorts. Due to its apical growth, in longer thalli, the apical region represents an increasingly smaller portion of biomass, resulting in different metabolic rates between these classes. Each sample was then measured and grouped as described, matching with the size class they fitted in. This classification was executed, with the work of Carballeira et al. as its basis, where the method of using thallus length as a proxy of thallus age is described and analyzed [25].

Each size class sample was then subdivided according to their genders by observation through a magnifying hand lens or a microscope: (1) Manual transverse sections of the receptacles were cut and observed to identify the presence of the large, dark oogonia, indicative of mature female conceptacles/fronds, which were separated. Then, (2) to differentiate between non-mature females and males, the same type of transverse sections was performed and observed in the microscope, at 40× magnification, to positively identify the presence of male reproductive organs, the antheridiophores. Images of males and females similar to those obtained in this study are shown in the following link: https://www.seaweed.ie/algae/fucus.php (accessed on 1 November 2021). During the collection of samples, they were kept at 4 °C and analyzed within 1 h. All samples, now grouped by size class and sex, were stored at −20 °C until lyophilization. Each sample class was represented by 7 replicates of thalli of their correspondent group.

### 2.2. Aqueous Extractions

Two types of aqueous extractions were performed: (1) decoction (100 °C) for 30 min; (2) 24 h extraction at 25 °C. For each extraction the entire thallus of all replicates was utilized and both types of extraction were made, obtaining 14 samples in total in a slightly modified protocol described in [26]. Shortly after, 5 g of the seaweed powder (ground in an electric food mill) was mixed with 100 mL of distilled water and incubated in a platform shaker for 24 h at 200 rpm at 25 °C. Decoction was prepared using 5 g of the seaweed powder boiled for 30 min in 100 mL of distilled water. Both types of mixtures were centrifuged at 3500× *g* for 10 min at 4 °C (JA-20 rotor using a Beckman^®®^ J2-21M/E Centrifuge, Beckman Coulter, Indianapolis, IN, USA). The supernatants of these extracts were then lyophilized and stored at −20 °C.

### 2.3. Total Phenolic Content Analysis

Total phenolic content (TPC) was measured spectrophotometrically using Folin–Ciocalteu reagent and phloroglucinol as the standard (calibration curve 10 to 1000 µg/mL). The concentration of total phenolic content was calculated as micrograms of phloroglucinol equivalents as the mean of the three replicates per milligram of dry seaweed extract (µg/mg DW), as described in [26].

### 2.4. Antioxidant Activity Determination

The antioxidant properties of all classified samples of *F. vesiculosus* were determined using 2,2-diphenyl-1-picrylhydrazyl (DPPH) radical scavenging assay to evaluate the capacity of *F. vesiculosus* to reduce the DPPH radical, following an adaptation of the DPPH method described by Falé et al. [27]. In this assay, a water blank was used. The percentage of antioxidant activity was obtained by using the following formula: AA (%) = [(A_sample_/A_control_) × (−1) +1] × 100, where AA (%) corresponds to the percentage of antioxidant activity, A_sample_, to the absorbance of each sample, and A control corresponds to the absorbance of the control solution.

### 2.5. Chemical Analysis by HPLC-DAD and LC-HRMS-MS/MS

Both types of extracts prepared were subjected to chromatographic analysis by conducting High-Performance Liquid Chromatography with Diode Array Detector (HPLC-DAD). The amount of 1 mg/mL of each extract was analyzed in a VWR-Hitachi Elite LaChrom^®®^ equipped with a LiChroCART^®®^RP-18, 5 μm, 250 × 4 mm, 100 Å column from Merck, autosampler L-2200, column oven L-2300 and diode array detector (DAD) L-2455. In this step, the mobile phase consisted of 0.05% (*v*/*v*) of trifluoracetic acid (Merck) in water and acetonitrile (Carlo Erba, Val-de-Reuil, France). Detection was carried out between 200 and 600 nm using diode array detector (DAD), and data acquisition was carried out by using EZChrom Elite^®®^ Hitachi Japan software.

LC/HRMS analysis was performed by liquid chromatography-high resolution tandem mass spectrometry (LC-HRMS/MS), as described in [26,27]. Acquired data were processed by DataAnalysis 4.1 software (Bruker Daltonik GmbH, Bremen, Germany). This identification was carried out by comparing the retention time as well as exact mass from the DataAnalysis^®®^ program version 4.4 from BRUKER, with results in [26].

### 2.6. Acetylcholinesterase Activity

An essay to determine *F. vesiculosus* extract inhibition towards acetylcholinesterase was carried out following a similar protocol described by Falé et al. (2013) [27]. An amount of 325 μL of 50 mM Tris buffer pH 8, 100 μL of sample and 25 μL acetylcholinesterase solution containing 0.26 U/mL were mixed in a spectrophotometer cell and allowed to incubate for 15 min at 25 °C. Subsequently, 75 μL of a solution of AChI (0.023 mg/mL) and 475 μL of 3 mM Ellmen’s reagent (DTNB) were added. The absorbance was read at 405 nm during the first 5 min of the reaction, and the initial velocity was calculated. A control reaction, which was considered to have 100% activity, was carried out using the same volume of water instead of sample. Percentage inhibition was calculated as follows

I (%) = 100 − (V_sample_/V_control_) × 100

where I is the percent inhibition of acetylcholinesterase, V_sample_ is the initial velocity of the reaction containing the extract and V_control_ is the initial velocity of the control reaction. The assays were carried out with at least l5 replicates, and a blank containing buffer instead of enzyme solution was used.

### 2.7. Heavy Metals Identification Essay

Minerals of dried *F. vesiculosus* samples were extracted by the digestion process and quantified using total X-ray fluorescence spectroscopy (TXRF) element analysis [28]. Acid mineralization was performed in Teflon reactors by adding 0.3 mL HClO4 and 1.7 mL HNO_3_ to approximately 0.2 mg of dried sample. The reactors were tightly closed and digestion occurred for 3 h in an oven at 110 °C [25]. After cooling, for each sample, 496 μL was recovered into 1.5 mL tubes, and 2 μL of Ga (final concentration 1 mg L^−1^) was added as the internal standard for mineral quantification. Samples were stored at 4 °C until analysis. Mineral quantification was determined by using total X-ray fluorescence spectroscopy (TXRF) element analysis. The cleaning and preparation of TXRF quartz glass sample carriers were performed, and 5 μL of the digested sample was added to the center of the carrier [28]. The carriers with the samples were aligned in a support containing a carrier with arsenic for gain correction (mono-element standard, Bruker Nano GmbH, Karlsruhe, Germany); a carrier with a nickel standard for sensitivity and detection limit (mono-element standard, Bruker Nano GmbH, Karlsruhe, Germany); and a carrier with a multi-element kraft for quantification accuracy (Kraft 10, Bruker Nano GmbH, Karlsruhe, Germany). Element measurements were carried out in a portable benchtop TXRF instrument (S2 PICOFOX™ spectrometer, Bruker Nano GmbH) for 800 s per sample. The accuracy and precision of the analytical methodology for elemental determinations were assessed by replicate analysis of certified reference material (ERM-CD200). Blanks and concurrent analysis of the standard reference material were used to detect possible losses/contamination during analysis. TXRF spectra and data evaluation interpretation were accomplished by using the Spectra 7.8.2.0 software (Bruker Nano GmbH, Berlin, Germany).

### 2.8. Statistical Analyses

Results were expressed in terms of mean ± standard error of the mean (SEM). Statistical comparisons were performed using a Three-Way ANOVA with Tukey’s HSD test on GraphPad 8.3.0. PCA, volcano test and clustering analysis were performed using statistic program MetaboScape^®®^ from Bruker.

## 3. Results and Discussion

### 3.1. Total Phenol Content

**Figure 1:**
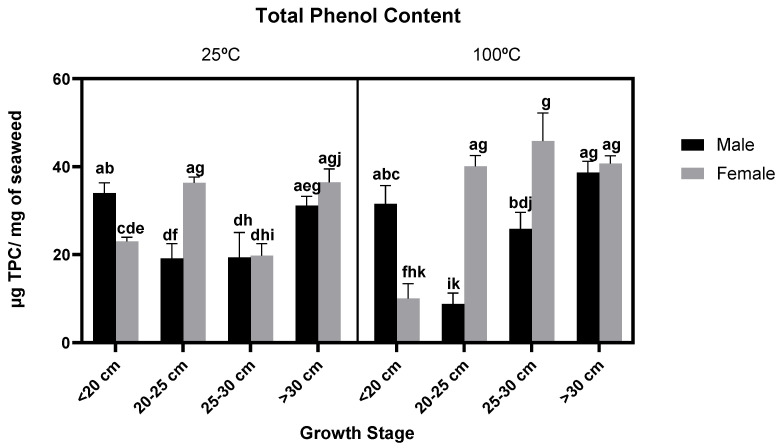
Total phenol content in a milligram of different *F. vesiculosus* extracts according to their gender, growth stage (µg TPC/mg of seaweed) and temperature of extraction (25 °C and 100 °C). Different superscript letters (a–j) correspond to values that can be considered statistically different (*p*-value ≤ 0.05). A three-way ANOVA was carried out for statistical analysis between gender, growth stage and temperature of extraction with Tukey’s HSD test for multiple comparisons represented by the superscripted letters.

The effect of *F. vesiculosus* gender and growth stage on the phenolic content within the thallus, using phloroglucinol as a standard, can be observed in Figure 1 (A and B, for extracts at 25 °C and 100 °C, respectively).

Total phenol content, measured as phloroglucinol equivalents, in general, achieved higher values in the longer thalli corresponding to older individuals. As TPC was previously shown [29] to be higher in vegetative thalli of this species, one can infer that higher vegetative thallus age can be indicative of higher TPC. Additionally, both the gender and growth stage of *F. vesiculosus* had a significance effect on the TPC (*p*-values < 0.0001 and *p*-value = 0.0079) as did the interaction of these two factors (*p*-value < 0.0001).

On the other hand, both types of extracts from younger males (<20 cm) achieved higher TPC, particularly in samples extracted at 100 °C in which there were significant differences between the two youngest male growths (Tukey’s HSD: <20 vs. 20–25 cm; *p*-value < 0.0001) and between the youngest male and female (Tukey’s HSD: male < 20 vs. female < 20 cm; *p*-value < 0.0001). These results may be indicative of a potential role played by phlorotannins in the early stages of *F. vesiculosus* development and sexual maturation. The tendency shown by TPC in both genders might also indicate that the role of phlorotannins is different regarding sexual maturation of the gametes within the conceptacle, as male gametes mature earlier and remain fertile for longer periods than female gametes.

Additionally, the temperature of extraction may influence TPC values—the temperature of extraction significantly affected the TPC (*p*-value < 0.0001)—as it was previously shown that in *F. vesiculosus* samples extracted at lower temperatures, particularly 25 °C, phlorotannin content was significantly lower when compared to samples extracted at higher temperatures [30].

### 3.2. Antioxidant Activity Determination

**Figure 2:**
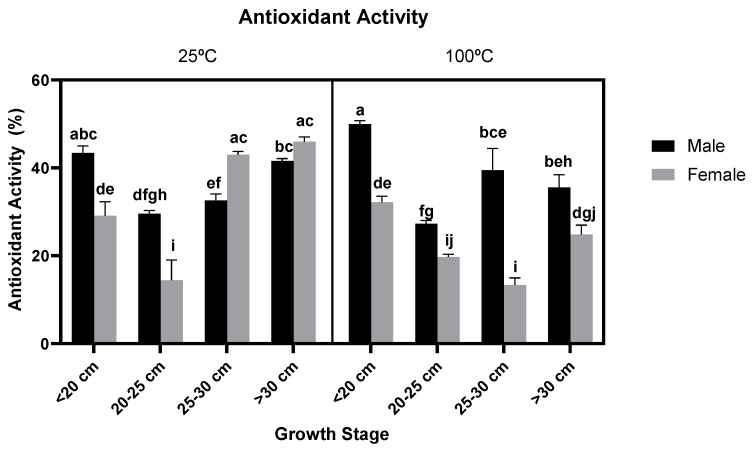
Antioxidant activity represented by the percentage of inhibition of DPPH radical of different *F. vesiculosus* extracts according to their gender, growth stage and temperature of extraction (25 °C and 100 °C). Different superscript letters (a–j) correspond to values that can be considered statistically different (*p*-value ≤ 0.05). A three-way ANOVA was carried out for the statistical analysis between gender, growth stage and temperature of extraction with Tukey’s HSD test for multiple comparisons, represented by the superscripted letters. In all tests, 0.1 mg/mL were used.

The effect of *Fucus vesiculosus* gender and growth stage on antioxidant activity within the seaweed can be observed in Figure 2 (A and B, for extracts at 25 °C and 100 °C, respectively).

In a similar pattern to the data obtained for the total phenolic content, for the extracts obtained at 100 °C extracts, the class represented by the youngest males (<20 cm) achieved significantly higher inhibition rates of DPPH radical scavenging when compared to all other male counterparts (*p*-values *<* 0.004). In a similar fashion to TPC, again the gender and age had a significant effect on antioxidant activity (*p*-values < 0.0001) as did their interaction (*p*-value *<* 0.0001). However, contrary to the TPC values, the inhibition rate experienced a decrease in most older stage extracts obtained at 100 °C. At this temperature of extraction, it is also noteworthy to mention that the youngest female thalli (<20 cm) also registered the highest antioxidant activity for its gender. These antioxidant activities were lower than that obtained with pure compounds such as BHT (butylated hydroxytoluene) or even other phenolic compounds such as quercetin and rutin, EC_50_ values of 16 μg/mL, 8 μg/mL and 12 μg/mL [31].

### 3.3. Acetylcholinesterase Activity

**Figure 3:**
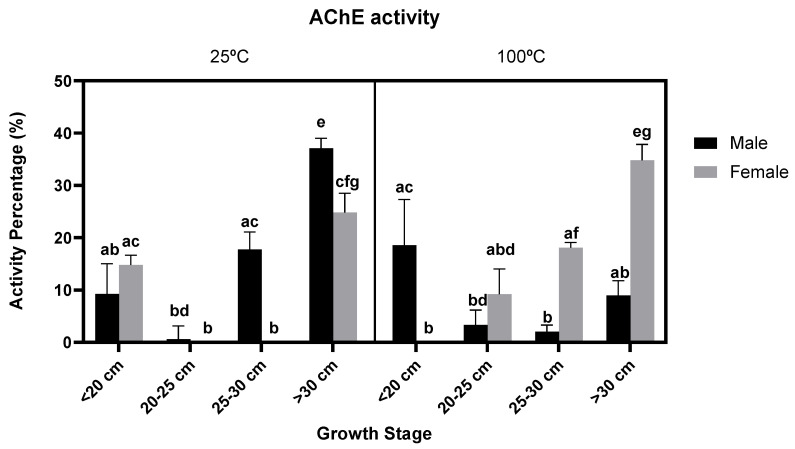
Effect on the AChE activity, percentage of inhibition of different *F. vesiculosus* extracts according to their gender, growth stage and temperature of extraction (25 °C and 100 °C at 1 mg/mL). Different superscript letters (a–g) correspond to values that can be considered statistically different (*p*-value ≤ 0.05). A three-way ANOVA was carried out for the statistical analysis between gender, growth stage and temperature of extraction with Tukey’s HSD test for multiple comparisons, represented by superscripted letters.

The effect of the different *F. vesiculosus* extracts on the AChE activity, across different growth stages and gender, is displayed in Figure 3 The results obtained showed that AChE activity, albeit being overall low, was greater in the older samples. Here, only the growth stage showed a significant effect on acetylcholinesterase activity (*p*-value < 0.0001). However, this assay was carried out without purification through solid-phase extraction (SPE), which could explain the low inhibitory activity towards AChE—the phlorotannin detected in this a seaweed can be too large to fit inside the AChE activity sites—as previous studies showed that this type of purification can result in better activities [26].

### 3.4. Characterization of Compounds Present in F. vesiculosus

In order to characterize the presence of different compounds or evaluate their variation throughout the development and gender of *F. vesiculosus* of the Tagus estuary, the chromatographic profiles of the compounds present in the different samples were obtained and compared.

#### 3.4.1. Characterization by High-Performance Liquid Chromatography with Diode Array Detector (HPLC-DAD)

**Figure 4:**
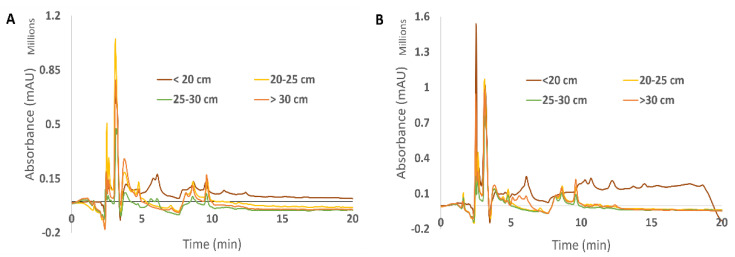
HPLC-DAD chromatographic profile of (**A**) male and (**B**) female samples of *F. vesiculosus,* extracted at 100 °C (1 mg/mL), across four growth stages—(-) <20 cm, (-) 20–25 cm, (-) 25–30 cm and (-) >30 cm. The y-axis was adjusted according to each respective maximum peak (1,200,000 mAU for (**A**) and 1,600,000 mAU for (**B**)).

**Figure 5:**
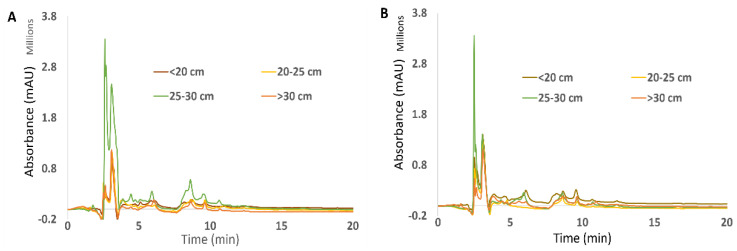
HPLC-DAD chromatographic profile of (**A**) male and (**B**) female samples of *F. vesiculosus*, extracted at 25 °C (1 mg/mL), across four growth stages—(-) <20 cm, (-) 20–25 cm, (-) 25–30 cm and (-) >30 cm. The y-axis was adjusted according to each respective maximum peak (3,800,000 for both chromatograph).

All extracts were analyzed by high-performance liquid chromatography with diode array detector (HPLC-DAD), and the chromatographic profiles of each growth stage within gender and type of extract were compared, displayed in Figure 4 and Figure 5.

The peaks with maximum intensities in all analyses were detected at around the 3 min mark retention time, exhibiting, however, differences between them. For instance, in the samples extracted at 100 °C, there was one major peak at the 2.50 min mark, with a peak intensity of 1,631,062 mAU detected in the females corresponding to the <20 cm growth stage—when compared to the male counterpart, the peak intensity for the same retention time is 232,675 mAU. In addition, for the second type of extracts analyzed (25 °C), there were two major peaks observed in both females and males with thalli length of 25–30 cm. In particular, in the male specimens, a peak at 2.61 min retention time with a peak intensity of 3,588,842 mAU was detected, whereas at the same retention time in extracts obtained at 100 °C, the peak intensity is only 12,858 mAU. Furthermore, another peak at 3.06 min with a peak intensity of 1,298,068 mAU was detected, while the extracts from males obtained at 100 °C only registered a peak intensity of 55,220 mAU. In a similar fashion, as described, the female samples extracted at 25 °C followed a similar pattern. Two major peaks were detected—at 2.50 min retention time with a peak intensity of 3,512,568 mAU and another at 3.06 min retention time with a peak intensity of 1,073,991 mAU. However, for female samples extracted at 100 °C, these same peaks had different peak intensities—230,893 mAU and 573,396 mAU, respectively—for the retention times described.

These differences in peaks may be indicative that there are components important to the development of *F. vesiculosus* at an intermediate stage in its development (25–30 cm). Therefore, certain compounds may exist in different stages of *F. vesiculosus* and at different concentrations, which may have an influence on what role they play in the seaweed’s development. As retention times between genders are quite similar, it can be theorized that the same compounds appear in both female and male specimens of *F. vesiculosus.*

#### 3.4.2. Compound Identification by LC/HRMS-MS

**Figure 6:**
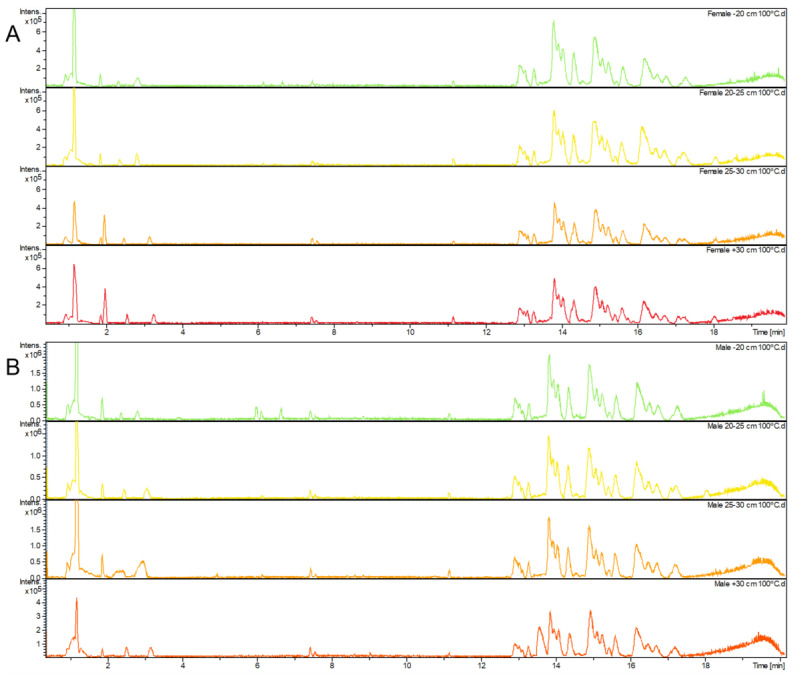
Chromatographic profiles of the compounds found across four growth stages (<20 cm, 20–25 cm, 25–30 cm and >30 cm) of female (**A**) and male (**B**) extracts of *F. vesiculosus*, obtained by using LC/HRMS-MS in ESI negative mode. Extracts carried out at 100 °C.

Although general chromatographic profiles of the samples throughout the growth stages and genders were similar, some major peaks differed in intensity between male and female specimens extracted at 100 °C. Furthermore, these extracts have shown, in general, better values for biological activities. As such, a tentative identification of the compounds present in the 100 °C extracts throughout the growth stages and genders was carried out by using liquid chromatography-high resolution tandem mass spectrometry (LC-HRMS/MS) in order to find any differences between them. The chromatograms were obtained in positive and negative modes, but only the negative mode is presented here (Figure 6).

In line with what was previously shown with the chromatograms obtained from RP-HPLC, the chromatographic profiles acquired with the usage of LC/HRMS-MS indicated very similar patterns between all growth stages and genders. In this aqueous extract, several phloroglucinols were identified; nevertheless, primary metabolites such as mannitol and citric acid were the most abundant ones. Subsequently, the identification of key compounds present in these extracts was carried out by comparing them to previously identified ones in different sets of *F. vesiculosus* samples from the Tagus estuary [26]. The identifications were carried out for 7 min where bigger differences seemed to exist in the chromatogram. The results were also previously obtained with different samples of this brown seaweed [26]. A heatmap representing the variation of the intensity of the compounds identified throughout gender and growth stages was also obtained, as described in Table 1.

In these extracts, phlorotannin was identified with compounds at retention time 1.9, but several phloroglucinol derivatives were identified, which confirms the positive values in the TPC test. Previous work on extracts from *F. vesiculosus* obtained from Tagus river estuary did not find many phlorotannins. Nevertheless, these were observed in seaweeds collected from the Atlantic Ocean [26].

**Figure 7:**
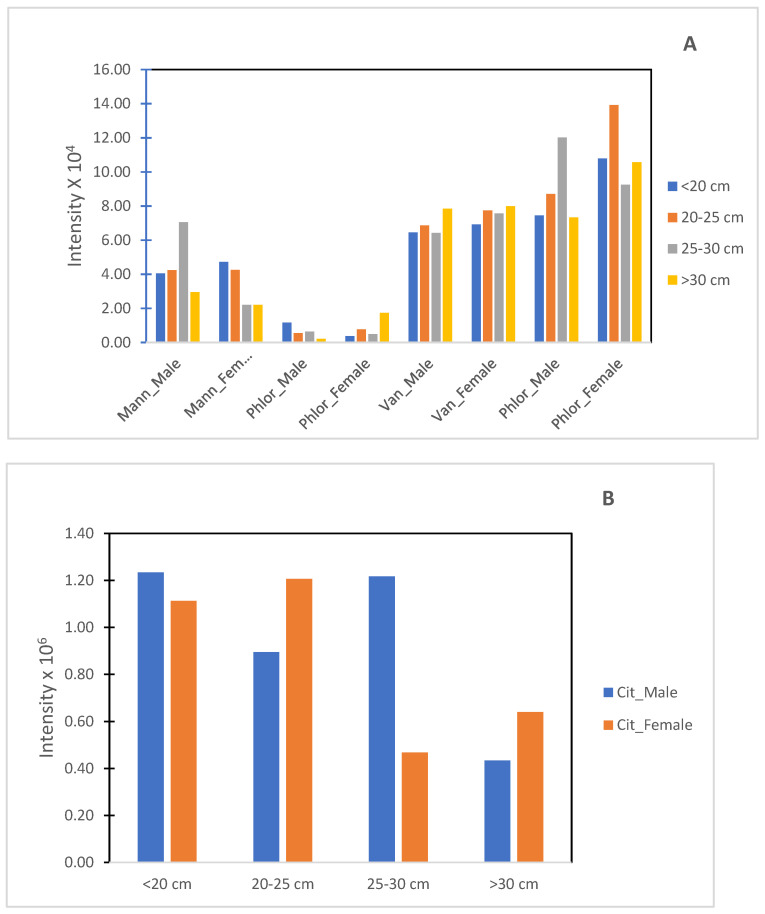
Evolution of metabolites relatively during growth. (**A**) Mannitol, vanillic acid and two phloroglucinols; (**B**) citric acid.

The evolution of each compound during seaweed growth, for males and females, can be observed in Figure 7.

**Figure 8:**
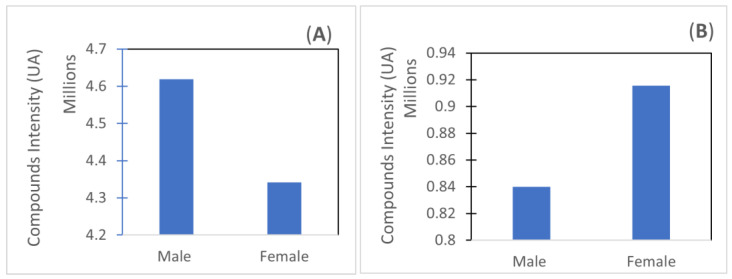
Total compounds intensity for male and female seaweed: (**A**) including citric acid; (**B**) not including citric acid.

In Figure 7A, the phloroglucinol derivative, at retention time 3.2, was not introduced since it only appeared in the late stages of growth. From Figure 7, it can be observed that there is no clear evolution of compounds during growth. Mannitol seems to decrease in female and vanillic acids keep constant during the growth of both genders (Figure 7A). Citric acid (Figure 7B) seems to decrease as seaweed ages. By adding the intensities of the identified compounds (Figure 8), it can be seen that male seaweeds had higher intensities when citric acid is taken into account (Figure 8A), which may mean a higher concentration of compounds. Females had higher intensities of compounds when discarding citric acid (Figure 8B). Compounds such as mannitol [32], citric acid [33,34] and phlorotannins [35] such as those found in the aqueous extract of *F. vesiculosus* have been described as having antioxidant activity.

The highest activities were dependent on seaweeds’ gender, and antioxidant activity was obtained for male seaweeds, while AChE activity was obtained for females. This seems to indicate that citric acid is important for antioxidant activity. The antioxidant activity of citric acid was already reported in several studies [33,34]. On what concerns the antioxidant activity, as the thallus is growing, phlorotannins may polymerize to more complex chains, whilst when the thallus is younger, they can remain in shorter oligomeric forms [29]. These lower weight phlorotannins may well be associated with higher antioxidant activity in *F. vesiculosus*, as suggested by Hermund et al. [36]. They proposed and demonstrated a tendency to decrease in antioxidant activity as the polymerization of phlorotannin increases [36,37]. Similarly, our results also demonstrated that a link can be established between these lower molecular weight phlorotannins and higher antioxidant activity, also corroborating with these results.

On the other side, the highest enzyme inhibitory activity may be ascribed to phlorotannins such as at a retention time of 1.9 min. Nevertheless, phloroglucinol by itself showed no inhibitory activity towards AChE [26], meaning that a higher structure will be more prone to fit inside the enzyme’s active site. AChE is inhibited by compounds having phenolic moieties in its structure due to the establishment of π–π interactions at the active site entrance [38], and this is the case with phlorotannins [26,39].

**Figure 9:**
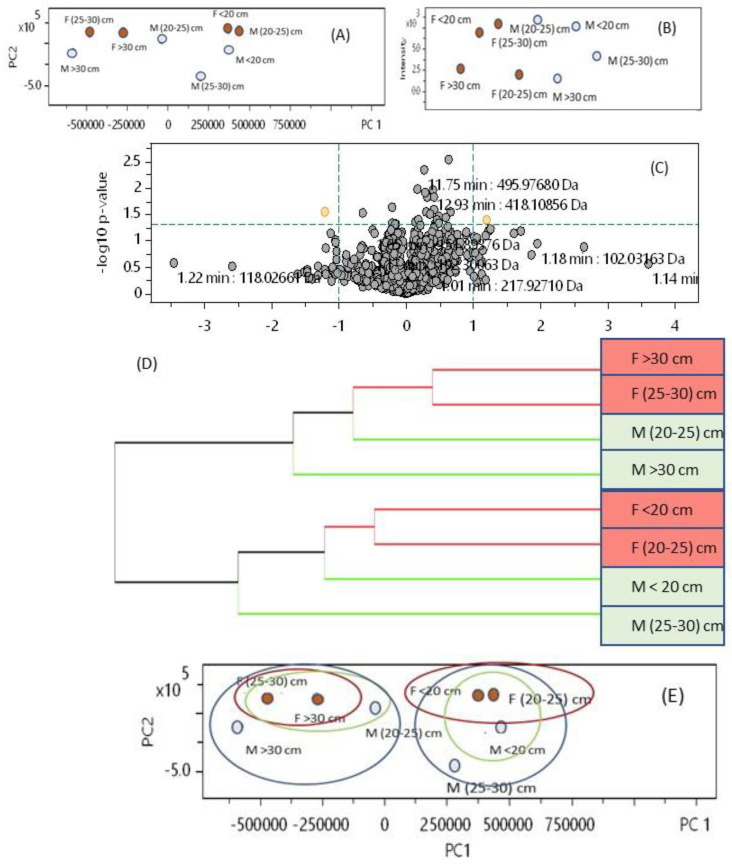
Statistical analysis of the peaks detected in the LC-HRMS analysis of female and male samples obtained at 100 °C: (**A**) PCA; (**B**) intensities; (**C**) volcano plot; (**D**) clustering; (**E**) PCA with groups assembled.

The comparison between males and females, at all stages of development, using the intensities of all the peaks detected by LC-HRMS and applying statistical analysis can be observed in Figure 9.

PCA analysis (Figure 9A) indicated that females and males cannot be grouped apart. Intensities of all peaks (Figure 9B) indicated that males and females presented the same order of intensities and no relationship with age could be noticed. The volcano plot (Figure 9C) revealed in yellow two compounds having *p* < 0.05, but they had a very low intensity that did not permit their identification. Clustering analysis (Figure 9D) indicated that females with thalli length 25–30 cm and >30 cm were similar, as were females in the <20 cm and 20–25 category. Therefore, females can be divided into two sub-groups—<25 cm and >25 cm. Males are more difficult to cluster, but they can be clustered with females at different ages (Figure 9E).

### 3.5. Elemental Analysis Using TXRF

The mineral composition of the extracts of *F. vesiculosus* was obtained by using elemental analysis using TXRF, and the elements found throughout the different growth stages and genders are described in Table 2. Their significance is expressed in microgram per gram of dry weight. The main minerals found in these samples were Na, Fe, K, Ca and Mg, which align with the constitution of this species through different studies in different habitats [3,40]. The main differences lay within the concentrations of the main minerals, which can vary due to a multitude of different factors, involving the growth conditions of each different individual, mainly attributed to the geographical location, mineral composition of the site, pH level, salinity and water temperature [41]. Moreover, endogenous factors such as cell wall and polysaccharide constitution can also affect the absorbance of these minerals [42].

Between each class, there were no significant differences between the concentrations of each mineral, except for the Na values, which may be attributed to any of the previously mentioned variation factors. However, since no significant differences were found between each growth stage and gender, such variance in Na values may be due to exogenous causes.

In addition, a comparison between various sources—Food and Drug Administration (FDA) [43], National Academy [44,45] and European Food Safety Authority (EFSA) [46]—for the recommended daily intake (RDI) of the minerals found in this essay and the concentrations detected in the extracts was carried out and presented in Table 3. The values of each mineral in *F. vesiculosus* adult male are presented on a 100 mg basis for a better comparison with international regulatory thresholds.

As evidenced, these samples from the Tagus Estuary can constitute good natural sources for different minerals, particularly the following: (i) Iodine, due to its high content, can be a good source for hypothyroidism treatment [47] since it is particularly important for the synthesis of thyroid hormone and, thus, the deficiency of this element may cause anomalies in the functioning of the thyroid [48]; (ii) chromium, particularly its trivalent form (Cr^3+^), for its prevalence in the treatment of type-2 diabetes can help improve blood sugar control, enhance the activity of insulin and, thus, help reduce cardiovascular diseases [49,50,51]; (iii) manganese, due to its function in bone growth as well as carbohydrate, lipid and amino acid metabolism [52]; and (iv) iron, which is an important element in nutrition, as iron insufficiency is one of the most common causes for anemia [53]. However, although these extracts may indicate a good potential as a good alternative natural source of various minerals, any type of supplementation should always be supervised, especially in individuals with a mineral deficiency.

Lastly, an evaluation on the presence and accumulation of heavy metals by the extracts of *F. vesiculosus* was carried out by comparing the values detected in the extracts to provisional tolerable weekly intakes (PTWI) and upper intake levels (UL) recommended, which can be observed in Table 4 [51,52,53].

As a result of heavy metal accumulation in estuaries and other marine environments, many types of seaweed are being studied as potential species for biomonitoring these areas and particularly for their capacity to bioaccumulate heavy metals [57,58]. Although these results suggested an absorption of certain heavy metals by *F. vesiculosus*, this type of experiment is usually performed together with an analysis of the heavy metals contents in the sediments [59,60], which vegetation or seaweed adhered to, in order to perform a proper evaluation of seaweed biomonitoring potential, as observed in a study by Duarte et al. (2021) [4]. These data also suggested that even though there are heavy metals present in the samples harvested, their concentrations did not reach nor surpass their respective upper intake levels for human consumption, according to the international guidelines thresholds. Moreover, this was commonplace throughout all others growth stage and genders samples; thus, only the >30 cm male specimen of *F. vesiculosus* was shown in the table, for ease of comparison. The samples from this Tagus estuary site also reported lower values of heavy metals, particularly Zn, Fe and Cu, while showing similar levels of Zn when compared to *F. vesiculosus* from the Humber estuary, England [61]. In addition, the levels for the mentioned metals, including Zn, were also lower in individuals of this species from the Menai Straits, Wales [62].

Furthermore, this may be possible due to the role of physodes within the thallus. These structures are commonly found in surface cells and have been described to potentially function as a first filter to stop heavy metals from gaining access to inner parts of the thallus [63]. At the same time, seaweeds cells simultaneously release the complexes of physodes that retain incoming heavy metals, thus cleansing the thallus from these compounds.

From a general standpoint, the youngest thalli (<20 cm), specifically the male specimen, seemed to achieve higher values regarding their biological activities, followed by the older growth stages (>30 cm), which are mostly independent of gender. Therefore, the question of which class should be harvested and used rises. This depends very much on the purpose of the work. On one hand, if it is to simply look at results and choose which growth stage/gender can produce the best activities, one would be pressed to choose males <20 cm. However, as differentiation between genders requires microscopic observation, this may not always be the best option in terms of practicality, especially if it involves large numbers of samples. Therefore, outside of studies in vitro and on a larger scale, using *F. vesiculosus* specimens with a thalli length > 30 cm may provide a better alternative, as these individuals still achieve high bioactivities, and processing does not require an extra step.

Regarding its consumption, the samples extracted at 100 °C, which mimic the preparation of a soup of *F. vesiculosus*, seemed to provide the best overall value.

## 4. Conclusions

*Fucus vesiculosus* L. from the Tagus estuary has a high value for its phenolic contents, particularly in the older females’ stages at 100 °C. The youngest stages also show high antioxidant activity, particularly for the males. Variation in components throughout *F. vesiculosus* growth stages also occurs, which may factor seaweeds’ development. Extracts from oldest males and females showed inhibitory activity towards AChE, although further studies of purified extracts should provide clearer results. This activity maybe to be due to phlorotannin presence. Soup preparation from dry seaweed was demonstrated as being an overall good source of various bioactive compounds, while not accumulating heavy metals in any harmful concentrations for consumption. Nevertheless, these aqueous extracts are a promising source of bioactive molecules that might be further explored by taking into account males of females and their ages at the moment of collection. In the moment of collecting *F. vesiculosus* at the river estuary and in the impossibility of distinguishing males from females, younger seaweeds seem to be preferred if antioxidant activity is the objective, and older ones are preferred if phloroglucinol compounds are the main focus for enzyme inhibition activity, for instance.

## Figures and Tables

**Table 1:** Tentative identification of several compounds present in aqueous extracts at 100 °C of *F. vesiculosus* and corresponding heatmap representing their intensity throughout growth stages and genders. M.: Male; F.: Female. Intensity: 1,200,000—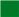; 1000—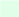. n.d: not detected.

			Intensities				
Rt (min)	Proposed Compound	Accurate [M-H] ^−^ *m*/*z* (Error, ppm)	<20 cm	20–25 cm	25–30 cm	>30 cm	
1.1	Mannitol	181.0719 (0.76)	40,486	42,480	70,480	29,590	M
			47,238	42,634	22,130	22,134	F
1.2	Citric Acid	191.0195 (−1.18)	1,233,346	894,690	1,217,112	434,158	M
			1,112,302	1,205,844	467,928	640,274	F
1.6	Glycidyl compound	551.1826 (−0.52)	1112	1070	9630	1752	M
			1764	2500	2164	2730	F
1.9	Phloroglucinol derivative (phlorotannin of fucol type)	497.0721 (−0.91)	11,740	5686	6402	2212	M
			3762	7770	4944	17,434	F
2	Glycosidic derivative	327.1315 (5.59)	2218	n.d	n.d	n.d	M
			n.d	1222	n.d	n.d	F
2.2	3,5,7-Trihydroxy-4-oxochromene-2-carboxylic acid	237.0074 (14.03)	n.d	n.d	n.d	n.d	M
			14,472	n.d	n.d	n.d	F
2.4	Vanillic acid sulfate	246.9916 (−0.8)	64,612	68,672	64,316	78,434	M
			69,226	77,480	75,676	79,882	F
3.2	Phloroglucinol glycosidic derivative	277.0926 (−1.05)	74,446	87,044	120,130	73,364	M
			107,880	139,220	92,550	105,762	F
3.2	Phloroglucinol glycosidic derivative	555.1742 (−0.72)	n.d	n.d	n.d	2600	M
			n.d	n.d	3352	6576	F
4.5	Tetrapeptide ProSerGlyPro	355.1615 (−2.28)	n.d	n.d	n.d	n.d	M
			n.d	1906	n.d	n.d	F
5	Butanedioic derivate	175.0554 (−33.11)	n.d	n.d	29,672	n.d	M
			n.d	n.d	n.d	n.d	F
6.7	Chroman-2,5-diol	165.0554 (−1.93)	87,114	n.d	n.d	n.d	M
			55,278	n.d	n.d	4506	F

**Table 2:** Mineral Composition (mg/100 g) of 6 different profiles of *F. vesiculosus*, comprising 4 growth stages and both genders. (*n* = 3). n.d: not determined.

	*F. vesiculosus* Growth Stage per Gender Analyzed					
	<20 cm F	20–25 cm M	25–30 cm F	25–30 cm M	>30 cm F	>30 cm M
Na	117.12 ± 13.22	185.57 ± 88.16	92.50 ± 11.85	165.84 ± 36.20	124.59 ± 42.27	158.73 ± 25.89
Mg	7.55 ± 1.03	8.62 ± 1.51	6.72 ± 2.78	10.57 ± 0.82	4.50 ± 0.60	9.54 ± 0.98
K	16.39 ± 14.30	13.38 ± 4.89	11.77 ± 3.38	16.10 ± 3.60	7.87 ± 2.92	12.91 ± 4.24
Ca	35.08 ± 20.20	26.63 ± 8.45	24.54 ± 3.88	31.00 ± 11.15	18.49 ± 3.41	26.14 ± 5.42
V	0.06 ± 0.02	0.09 ± 0.02	0.08 ± 0.04	0.08 ± 0.01	0.05 ± 0.01	0.07 ± 0.02
Cr	0.05 ± 0.02	0.08 ± 0.01	0.05 ± 0.01	0.08 ± 0.01	0.06 ± 0.01	0.10 ± 0.01
Mn	6.49 ± 1.66	10.91 ± 1.56	7.18 ± 0.63	11.64 ± 1.40	8.83 ± 0.70	10.97 ± 1.22
Fe	17.08 ± 4.21	24.70 ± 4.59	19.12 ± 2.56	28.68 ± 2.79	18.88 ± 4.33	28.27 ± 4.30
Co	0.14 ± 0.03	0.24 ± 0.06	0.18 ± 0.02	0.28 ± 0.05	0.20 ± 0.05	0.28 ± 0.05
Ni	0.08 ± 0.02	0.14 ± 0.02	0.09 ± 0.01	0.14 ± 0.02	0.11 ± 0.02	0.15 ± 0.01
Cu	0.26 ± 0.06	0.39 ± 0.07	0.23 ± 0.02	0.39 ± 0.07	0.30 ± 0.06	0.35 ± 0.03
Zn	2.79 ± 0.57	5.57 ± 0.47	3.18 ± 0.35	4.88 ± 0.60	3.44 ± 0.66	4.59 ± 0.27
As	0.21 ± 0.13	0.37 ± 0.19	0.23 ± 0.05	0.34 ± 0.07	0.29 ± 0.07	0.22 ± 0.02
Se	0.01 ± 0.00	0.01 ± 0.00	0.01 ± 0.00	0.01 ± 0.00	0.01 ± 0.00	0.01 ± 0.01
Br	2.55 ± 0.23	3.65 ± 0.85	2.71 ± 0.052	3.35 ± 0.52	2.69 ± 0.54	3.92 ± 1.19
Sr	19.22 ± 1.32	31.66 ± 6.60	20.60 ± 3.42	32.79 ± 11.28	22.47 ± 6.66	25.07 ± 2.15
Cd	n.d	n.d	n.d	n.d	n.d	n.d
I	0.99 ± 0.36	1.05 ± 0.36	1.13 ± 0.33	1.22 ± 0.38	0.74 ± 0.20	1.06 ± 0.10
Hg	0	0	0	0	0	0
Pb	0.74 ± 0.05	1.20 ± 0.40	0.72 ± 0.11	1.02 ± 0.22	0.71 ± 0.22	0.97 ± 0.15

**Table 3:** Comparison between various sources for the recommended daily intake (RDI) for essential minerals with the minerals detected in *F. vesiculosus*: FDA: Food and Drug Administration; EFSA: European Food Safety Authority; M: Male; W: Women; n.a.: not available.

	Mineral Recommended Daily Intake (RDI) vs. *F. vesiculosus* Mineral Composition			
	FDA [43]	National Academy [44,45]	EFSA [46]	>30 cm M
Na	2400 mg	2300 mg	2000 mg	158.73 mg
Mg	400 mg	M: 420 mg; W: 380 mg	M: 350 mg; W: 300 mg	9.54 mg
K	3500 mg	4700 mg	3500 mg	12.91 mg
Ca	1000 mg	1000 mg	1000 mg	26.14 mg
Cr	120 µg	M: 35 µg; W: 25 µg	n.a.	100 µg
Mn	2 mg	M: 2.3 mg; W: 1.8 mg	3 mg	10.97 mg
Fe	18 mg	M: 8 mg; W: 18 mg	M: 11 µg; W: 16 µg	28.27 mg
Cu	2 mg	900 µg	M: 1.6 mg; W: 1.5 mg	35 µg
Zn	15 mg	M: 11 mg; W: 8 mg	M:16.3 mg; W:12.7 mg	4.59 mg
Se	70 µg	55 µg	70 µg	10 µg
I	150 µg	150 µg	150 µg	1.06 mg

**Table 4:** Heavy metals detected in *F. vesiculosus* compared against tolerable intakes defined by EFSA and WHO. b.w.: body weight; UL: Upper Intake Level.

	Heavy Metals Tolerable Daily Intake (TDI) [45,54,55,56] vs. *F. vesiculosus* Mineral Composition	
		>30 cm M
Ni	2.8 µg/kg b.w.	150 µg
As	3 µg/kg b.w.	220 µg
Pb	25 µg/kg b.w.	970 µg
Fe	45 mg/day UL	28.27 mg
Zn	40 mg/day UL	4.59 mg
Cu	10 mg/day UL	35 µg

## Data Availability

Not applicable.

## References

[1] Houghton P.J. (1995). The Role of Plants in Traditional Medicine. J. Altern. Complementary Med..

[2] Cairrão E., Pereira M.J., Pastorinho M.R., Morgado F., Soares A.M.V.M., Guilhermino L. (2007). *Fucus* Spp. as a Mercury Contamination Bioindicator in Costal Areas (Northwestern Portugal). Bull. Environ. Contam. Toxicol..

[3] Lorenzo J.M., Agregán R., Munekata P.E.S., Franco D., Carballo J., Şahin S., Lacomba R., Barba F.J. (2017). Proximate Composition and Nutritional Value of Three Macroalgae: *Ascophyllum nodosum*, *Fucus vesiculosus* and *Bifurcaria bifurcata*. Mar. Drugs.

[4] Duarte B., Carreiras J., Feijão E., Reis-Santos P., Caçador I., Matos A.R., Fonseca V.F. (2021). Fatty Acid Profiles of Estuarine Macroalgae Are Biomarkers of Anthropogenic Pressures: Development and Application of 07a Multivariate Pressure Index. Sci. Total Environ..

[5] Rupérez P. (2002). Mineral Content of Edible Marine Seaweeds. Food Chem..

[6] Rupérez P., Ahrazem O., Leal J.A. (2002). Potential Antioxidant Capacity of Sulfated Polysaccharides from the Edible Marine Brown Seaweed *Fucus vesiculosus*. J. Agric. Food Chem..

[7] Imbs T.I., Zvyagintseva T.N. (2018). Phlorotannins Are Polyphenolic Metabolites of Brown Algae. Russ. J. Mar. Biol..

[8] Shibata T., Ishimaru K., Kawaguchi S., Yoshikawa H., Hama Y. (2008). Antioxidant Activities of Phlorotannins Isolated from Japanese Laminariaceae. J. Appl. Phycol..

[9] Kurutas E.B. (2016). The Importance of Antioxidants Which Play the Role in Cellular Response against Oxidative/Nitrosative Stress: Current State. Nutr. J..

[10] Valko M., Izakovic M., Mazur M., Rhodes C.J., Telser J. (2004). Role of Oxygen Radicals in DNA Damage and Cancer Incidence. Mol. Cell. Biochem..

[11] Pham-Huy L.A., He H., Pham-Huy C. (2008). Free Radicals, Antioxidants in Disease and Health. Int. J. Biomed. Sci..

[12] Halliwell B. (2001). Role of Free Radicals in the Neurodegenerative Diseases. Drugs Aging.

[13] Singh R., Sharad S., Kapur S. (2004). Free Radicals and Oxidative Stress in Neurodegenerative Diseases: Relevance of Dietary Antioxidants. J. Indian Acad. Clin. Med..

[14] MacNee W. (2001). Oxidative Stress and Lung Inflammation in Airways Disease. Eur. J. Pharmacol..

[15] Hoshino Y., Mishima M. (2008). Redox-Based Therapeutics for Lung Diseases. Antioxid. Redox Signal..

[16] Sung M.K., Bae S.C., Watson R., Preedy V. (2013). Dietary Antioxidants and Rheumatoid Arthritis. Bioactive Food as Interventions for Arthritis and Related Inflammatory Diseases.

[17] Dröge W. (2002). Free Radicals in the Physiological Control of Cell Function. Physiol. Rev..

[18] Meyer C.H., Sekundo W. (2005). Nutritional Supplementation to Prevent Cataract Formation. Dev. Ophthalmol..

[19] Villemagne V.L., Burnham S., Bourgeat P., Brown B., Ellis K.A., Salvado O., Szoeke C., Macaulay S.L., Martins R., Maruff P. (2013). Amyloid β Deposition, Neurodegeneration, and Cognitive Decline in Sporadic Alzheimer’s Disease: A Prospective Cohort Study. Lancet Neurol..

[20] Olasehinde T.A., Mabinya L.V., Olaniran A.O., Okoh A.I. (2019). Chemical Characterization of Sulfated Polysaccharides from *Gracilaria gracilis* and *Ulva lactuca* and Their Radical Scavenging, Metal Chelating, and Cholinesterase Inhibitory Activities. Int. J. Food Prop..

[21] Olasehinde T.A., Olaniran A.O., Okoh A.I. (2019). Aqueous–Ethanol Extracts of Some South African Seaweeds Inhibit Beta-Amyloid Aggregation, Cholinesterases, and Beta-Secretase Activities in Vitro. J. Food Biochem..

[22] Pangestuti R., Kim S.K. (2010). Neuroprotective Properties of Chitosan and Its Derivatives. Mar. Drugs.

[23] Francis P.T. (2005). The Interplay of Neurotransmitters in Alzheimer’s Disease. CNS Spectr..

[24] Brinza L., Nygård C.A., Dring M.J., Gavrilescu M., Benning L.G. (2009). Bioresource Technology Cadmium Tolerance and Adsorption by the Marine Brown Alga *Fucus vesiculosus* from the Irish Sea and the Bothnian Sea. Bioresour. Technol..

[25] Carballeira C., Rey-Asensio A., Carballeira A. (2014). Interannual Changes in Δ15N Values in *Fucus vesiculosus* L.. Mar. Pollut. Bull..

[26] André R., Guedes L., Melo R., Ascens L., Pacheco R., Vaz P.D., Serralheiro M.L. (2020). Effect of Food Preparations on In Vitro Bioactivities and Chemical Components of *Fucus vesiculosus*. Foods.

[27] Falé P.L., Ferreira C., Rodrigues A.M., Cleto P., Madeira P.J.A., Florêncio M.H., Frazão F.N., Serralheiro M.L.M. (2013). Antioxidant and Anti-Acetylcholinesterase Activity of Commercially Available Medicinal Infusions after in vitro Gastrointestinal Digestion. J. Med. Plants Res..

[28] Cruz de Carvalho R., Feijão E., Kletschkus E., Marques J.C., Reis-Santos P., Fonseca V.F., Papenbrock J., Caçador I., Duarte B. (2020). Halophyte Bio-Optical Phenotyping: A Multivariate Photochemical Pressure Index (Multi-PPI) to Classify Salt Marsh Anthropogenic Pressures Levels. Ecol. Indic..

[29] Tuomi J., Ilvessalo H., Niemela P., Siren S., Jormalainen V. (1989). Within-Plant Variation in Phenolic Content and Toughness of the Brown Alga *Fucus vesiculosus* L.. Bot. Mar..

[30] Ferreira R.M., Ribeiro A.R., Patinha C., Silva A.M.S., Cardoso S.M., Costa R. (2019). Water Extraction Kinetics of Bioactive Compounds of *Fucus vesiculosus*. Molecules.

[31] Henriques J., Falé P.L., Pacheco R., Florêncio M.H., Serralheiro M.L. (2018). Phenolic Compounds from Actinidia Deliciosa Leaves: Caco-2 Permeability, Enzyme Inhibitory Activity and Cell Protein Profile Studies. J. King Saud Univ. Sci..

[32] André P., Villain F. (2017). Free Radical Scavenging Properties of Mannitol and Its Role as a Constituent of Hyaluronic Acid Fillers: A Literature Review. Int. J. Cosmet. Sci..

[33] Ryan E.M., Duryee M.J., Hollins A., Dover S.K., Pirruccello S., Sayles H., Real K.D., Hunter C.D., Thiele G.M., Mikuls T.R. (2019). Antioxidant Properties of Citric Acid Interfere with the Uricase-Based Measurement of Circulating Uric Acid. J. Pharm. Biomed. Anal..

[34] Liu Q., Tang G.Y., Zhao C.N., Gan R.Y., Li H.B. (2019). Antioxidant Activities, Phenolic Profiles, and Organic Acid Contents of Fruit Vinegars. Antioxidants.

[35] Sathya R., Kanaga N., Sankar P., Jeeva S. (2017). Antioxidant Properties of Phlorotannins from Brown Seaweed *Cystoseira trinodis* (Forsskål) C. Agardh. Arab. J. Chem..

[36] Hermund D.B., Plaza M., Turner C., Jónsdóttir R., Kristinsson H.G., Jacobsen C., Nielsen K.F. (2018). Structure Dependent Antioxidant Capacity of Phlorotannins from Icelandic *Fucus vesiculosus* by UHPLC-DAD-ECD-QTOFMS. Food Chem..

[37] Catarino M.D., Silva A.M.S., Cardoso S.M. (2018). Phycochemical Constituents and Biological Activities of *Fucus* spp.. Mar. Drugs.

[38] Jang C., Yadav D.K., Subedi L., Venkatesan R., Venkanna A., Afzal S., Lee E., Yoo J., Ji E., Kim S.Y. (2018). Identification of Novel Acetylcholinesterase Inhibitors Designed by Pharmacophore-Based Virtual Screening, Molecular Docking and Bioassay. Sci. Rep..

[39] Kannan R.R.R., Aderogba M.A., Ndhlala A.R., Stirk W.A., van Staden J. (2013). Acetylcholinesterase Inhibitory Activity of Phlorotannins Isolated from the Brown Alga, *Ecklonia maxima* (Osbeck) Papenfuss. Food Res. Int..

[40] Balina K., Romagnoli F., Blumberga D. (2016). Chemical Composition and Potential Use of *Fucus vesiculosus* from Gulf of Riga. Energy Procedia.

[41] Peinado I., Girón J., Koutsidis G., Ames J.M. (2014). Chemical Composition, Antioxidant Activity and Sensory Evaluation of Five Different Species of Brown Edible Seaweeds. Food Res. Int..

[42] Peng Y., Hu J., Yang B., Lin X., Zhou X., Yang X., Liu Y. (2015). Chemical Composition of Seaweeds.

[43] FDA (2016). FDA Vitamins and Minerals Chart.

[44] Ross A.C., Taylor C.L., Yaktine A.L., Valle H.B. (2011). Del DRI Calcium Vitamin D.

[45] National Academies Press (2000). Dietary Reference Intakes for Vitamin A, Vitamin K, Arsenic, Boron, Chromium, Copper, Iodine, Iron, Mangenese, Molybdenum, Nickel, Silicon, Vanadium and Zinc.

[46] EFSA (2017). European Food Safety Authority Dietary Reference Values for Nutrients Summary Report. EFSA Supporting Publ..

[47] Zava T.T., Zava D.T. (2011). Assessment of Japanese Iodine Intake Based on Seaweed Consumption in Japan: A Literature-Based Analysis. Thyroid Res..

[48] Bove M., Stansbury J.E., Romm A. (2010). Endocrine Disorders and Adrenal Support. Botanical Medicine for Women’s Health.

[49] Lefavi R.G., Anderson R.A., Keith R.E., Wilson G.D., McMillan J.L., Stone M.H. (1992). Efficacy of Chromium Supplementation in Athletes: Emphasis on Anabolism. Int. J. Sport Nutr..

[50] Balk E., Tatsioni A., Lichtenstein A. (2007). Effect of Chromium Supplementation on A Systematic Review of Randomized Controlled Trials. Diabetes Care.

[51] Pechova A., Pavlata L. (2007). Chromium as an Essential Nutrient: A Review. Vet. Med..

[52] Wood R.J. (2009). Manganese and Birth Outcome. Nutr. Rev..

[53] Abbaspour N., Hurrell R., Kelishadi R. (2014). Review on Iron and Its Importance for Human Health. J. Res. Med. Sci..

[54] (1975). FAO/WHO Expert Consultation Evaluation of Certain Food Additives. World Health Organ.-Tech. Rep. Ser..

[55] World Health Organization (2019). Preventing Disease through Healthy Environments: Exposure to Arsenic: A Major Public Health Concern. https://apps.who.int/iris/handle/10665/329482.

[56] (2015). (EFSA) European Food Safety Authority Scientific Opinion on the Risks to Public Health Related to the Presence of Nickel in Food and Drinking Water. EFSA J..

[57] Sandau E., Sandau P., Pulz O., Zimmermann M. (1996). Heavy Metal Sorption by Marine Algae and Algal By-Products. Acta Biotechnol..

[58] Sáez C.A., Lobos M.G., Macaya E.C., Oliva D., Quiroz W., Brown M.T. (2012). Variation in Patterns of Metal Accumulation in Thallus Parts of *Lessonia trabeculata* (Laminariales; Phaeophyceae): Implications for Biomonitoring. PLoS ONE.

[59] Caçador I., Vale C., Catarino F. (1996). The Influence of Plants on Concentration and Fractionation of Zn, Pb, and Cu in Salt Marsh Sediments (Tagus Estuary, Portugal). J. Aquat. Ecosyst. Stress Recovery.

[60] Barreiro R., Picado L., Real C. (2002). Biomonitoring Heavy Metals in Estuaries: A Field Comparison of Two Brown Algae Species Inhabiting Upper Estuarine Reaches. Environ. Monit. Assess..

[61] Barnett B.E., Ashcroft C.R. (1985). Heavy Metals in *Fucus vesiculosus* in the Humber Estuary. Environ. Pollut. B Chem. Phys..

[62] Foster P. (1976). Concentrations and Concentration Factors of Heavy Metals in Brown Algae. Environ. Pollut..

[63] Schoenwaelder M.E.A. (2002). The Occurrence and Cellular Significance of Physodes in Brown Algae. Phycologia.

